# A-to-I mRNA editing recodes hundreds of genes in dozens of species and produces endogenous protein isoforms in bacteria

**DOI:** 10.1093/nar/gkaf656

**Published:** 2025-07-12

**Authors:** Eyal Elias, Isaac Gifford, Liron Didi, Ofir Fargeon, Danielle Arad, Rinat Cohen-Pavon, Gil Sorek, Liron Levin, Dganit Melamed, Liam Aspit, Jeffrey E Barrick, Dan Bar-Yaacov

**Affiliations:** The Shraga Segal Department of Microbiology, Immunology, and Genetics, Ben-Gurion University of the Negev, Beer-Sheva 8410501, Israel; Department of Molecular Biosciences, The University of Texas at Austin, Austin, TX 78751, United States; The Shraga Segal Department of Microbiology, Immunology, and Genetics, Ben-Gurion University of the Negev, Beer-Sheva 8410501, Israel; The Shraga Segal Department of Microbiology, Immunology, and Genetics, Ben-Gurion University of the Negev, Beer-Sheva 8410501, Israel; The Shraga Segal Department of Microbiology, Immunology, and Genetics, Ben-Gurion University of the Negev, Beer-Sheva 8410501, Israel; The Shraga Segal Department of Microbiology, Immunology, and Genetics, Ben-Gurion University of the Negev, Beer-Sheva 8410501, Israel; Department of Life Sciences, Ben-Gurion University of the Negev, Beer-Sheva 8410501, Israel; Bioinformatics Core Facility, llse Katz Institute for Nanoscale Science and Technology, Ben-Gurion University of the Negev, Beer-Sheva, 8410501 Israel; The Smoler Protein Research Center, Technion Israel Institute of Technology, Haifa 3200003, Israel; The Shraga Segal Department of Microbiology, Immunology, and Genetics, Ben-Gurion University of the Negev, Beer-Sheva 8410501, Israel; Department of Molecular Biosciences, The University of Texas at Austin, Austin, TX 78751, United States; The Shraga Segal Department of Microbiology, Immunology, and Genetics, Ben-Gurion University of the Negev, Beer-Sheva 8410501, Israel

## Abstract

Adenosine-to-inosine (A-to-I) messenger RNA (mRNA) editing can affect the sequence and function of translated proteins and has been extensively investigated in eukaryotes. However, the prevalence of A-to-I mRNA editing in bacteria, its governing regulatory principles, and its biological significance are poorly understood. Here, we show that A-to-I mRNA editing occurs in hundreds of transcripts across dozens of gammaproteobacterial species, with most edits predicted to recode protein sequences. Furthermore, we reveal conserved regulatory determinants controlling editing across gammaproteobacterial species. Using *Acinetobacter baylyi* as a model, we show that mutating TadA, the mediating enzyme, reduces editing across all sites. Conversely, overexpressing TadA resulted in the editing of >300 transcripts, attesting to the editing potential of TadA. Notably, we show for the first time, at the protein level, that normal levels of A-to-I mRNA editing lead to wild-type bacteria expressing two protein isoforms from a single gene. Finally, we show that a TadA mutant with deficient editing activity does not grow at high temperatures, suggesting that RNA editing has a functional role in bacteria. Our work reveals that A-to-I mRNA editing in bacteria is widespread and has the potential to reshape the bacterial transcriptome and proteome.

## Graphical Abstract

**Graphical Abstract:**
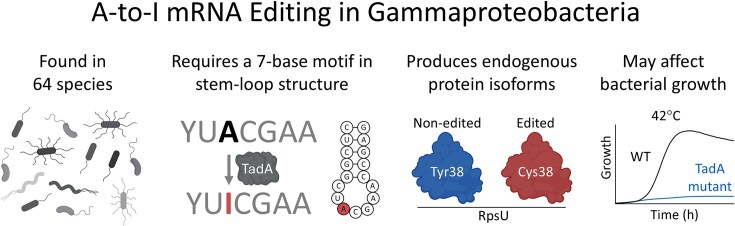

## Introduction

Adenosine-to-inosine (A-to-I) messenger RNA (mRNA) editing can affect the sequence and the function of translated proteins because the ribosome identifies inosine as guanosine [1–5]. A-to-I mRNA editing occurs in all major groups of multicellular organisms (metazoa) by enzymes that belong to the adenosine deaminase acting on RNA (ADAR) family [1, 3–11]. A-to-I mRNA editing is essential for preventing an aberrant double-stranded RNA immune response, crucial for proper neuronal activity, plays a role in cancer progression, affects embryonic development, and diversifies the proteome [10, 12–30]. Furthermore, A-to-I mRNA editing has been reported in fungi, even though they lack an ADAR homolog, supporting the occurrence of editing across eukaryotes [31–34]. In contrast, until recently, bacteria were thought to lack mRNA editing.

Previously, we demonstrated that mRNA editing occurs in bacteria (*Escherichia coli*) [35]. Nearly all mRNA editing events were A-to-I mRNA editing events (hereafter “mRNA editing,” or simply “editing” events), and all of these occurred within a TACG (UACG) motif—the same motif that is required for editing of the arginine transfer RNA (*tRNA^Arg2^*) anticodon by the enzyme tRNA-specific adenosine deaminase (TadA) [36]. This finding was surprising since TadA was thought to target tRNA exclusively [36–40]. By mutating and overexpressing TadA in *E. coli*, we demonstrated that TadA can edit mRNAs in addition to *tRNA^Arg2^* [35]. Remarkably, overexpression of TadA in *E. coli* led to the editing of hundreds of mRNAs, and most of these editing events are predicted to alter the protein sequences they encode. In agreement with our findings, it was recently shown that a homolog of TadA mediates A-to-I mRNA editing in fungi [41] and the bacterium *Streptococcus pyogenes* [42]. Furthermore, A-to-I mRNA editing was also reported to occur in mRNAs harboring the motif of TadA in *Klebsiella pneumoniae* [43]. However, the prevalence of mRNA editing across bacterial species, the regulatory principles governing its formation, and its ability to produce endogenously expressed bacterial protein isoforms are poorly understood.

## Materials and methods

### RNA editing discovery in gammaproteobacterial species

We downloaded data for 554 RNA-sequencing (RNA-seq) experiments with 96 different bacterial species (Supplementary Tables S1 and S2). We analyzed at least two biological samples in each species, while in most cases (92.7%), we analyzed three or more samples when available. All RNA-seq datasets had at least 557 206 350 sequenced bases. We used CLC Genomics Workbench for all steps of the analysis.

RNA-seq reads were first trimmed according to length and quality scores to ensure high quality of the reads by using the following parameters: quality limit = 0.05; trim ambiguous nucleotides = yes; maximum number of ambiguities = 2; automatic read-through adapter trimming = yes; and minimum length = 50.

Next, RNA-seq reads were mapped to the closest available reference genome with the following parameters: masking mode = no masking; match score = 1; mismatch cost = 2; cost of insertions and deletions = linear gap cost; insertion cost = 3; deletion cost = 3; length fraction = 0.95; similarity fraction = 0.95; global alignment = no; auto-detect paired distances = yes; and nonspecific match handling = ignore.

Downstream analysis was conducted only on samples with at least 50% reads mapped to their corresponding genome (belonging to 72 species).

Next, initial variant calling was performed using the following parameters: ignore positions with coverage above = 1 000 000; minimum coverage = 20; minimum count = 3; minimum frequency (%) = 1.0; base quality filter = yes; neighborhood radius = 2; minimum central quality = 30; and minimum neighborhood quality = 30.

After the initial variant calling was performed, additional filtering was applied: number of unique start positions ≥3; number of unique end positions ≥3; frequency ≥5%; variant frequency (putative editing level) ≤98 (to exclude strain-specific mutations); only two bases per position are allowed (not hyper allelic).

Next, we filtered for variants shared between 100% of biological replicates of each experiment from the same study.

Finally, we focused only on sites found in known genes to correct for sequence direction and to detect only A-to-G mismatches (and not T-to-C). After this step, we identified 1751 A>G mismatches (Supplementary Table S3).

**Figure 1.**
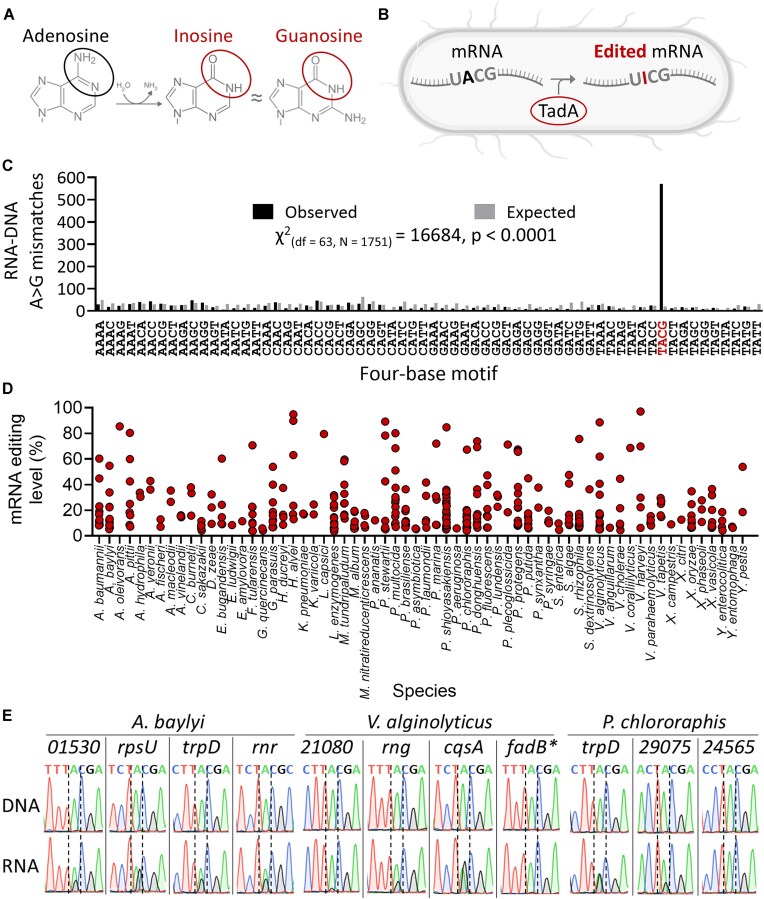
Hundreds of mRNAs are edited and predicted to recode protein sequences across 64 gammaproteobacterial species. (**A**) Adenosine is deaminated to inosine, which is similar to guanosine in its base-pairing properties. Therefore, ribosomes and reverse transcriptases recognize inosine as guanosine. (**B**) TadA is the only known mRNA (and tRNA) A-to-I editing enzyme in bacteria. TadA requires a TACG (UACG) motif in the targeted RNA for its activity. (**C**) Four-base motif distribution around A>G mismatches (possible RNA editing sites) between RNA-seq data and the reference genome of 72 bacterial species. We found significant enrichment for the TACG motif (*P*-value <.0001; chi-square test for goodness of fit; marked in red). In black is the observed distribution, and in gray is the expected motif distribution of 1751 A>G sites when sampling randomly from the genome of the examined species. (**D**) Distribution and average editing level (percent of edited transcript) of the 381 mRNA editing events across 64 gammaproteobacterial species with detected editing. (**E**) Sanger sequencing of matched DNA and RNA samples of 11 genes/transcripts in three representative species. We observed a double peak of A and G (I) in 10/11 examined sites in mRNA (complementary DNA, cDNA) sequences but not the corresponding DNA (genome) sequences. Above each site is the gene’s name or locus tag. *Editing is present as a small peak. See Supplementary Fig. S4 for a closeup.

See also Supplementary Figs S1 and S2 for the RNA editing discovery pipeline.

In some species, we downloaded samples from different experiments. Because of our filter parameters applied above (e.g. variants must be found in 100% biological replicates and with variant frequency above 5%), it is logical to assume that sites could be identified in samples from one experiment but not another. Thus, we used the list of the 1751 A>G variants and extracted variants from our initial variants analysis (variant frequency above 1%). This allowed us to identify A>G sites with variant frequencies between 1% and 100% across all samples and predict their effect on protein sequence (Supplementary Tables S4–S6).

### Identification of TadA homologs across bacterial strains

We used the *E. coli* TadA sequence as query and used (local) tBLASTn to search against the genomes of the tested species [44] (Supplementary Table S7).

### RNA editing evolutionary conservation analysis

In order to understand the level of conservation of editing events, we aimed to characterize the amino acid identity across gammaproteobacterial species concerning conserved editing events (Supplementary Table S8). We used the identified protein product accession of conserved editing events as a query in BLASTP analysis against up to 5000 gammaproteobacteria targets (the maximum allowed number of targets in NCBI’s web-blast) with an *e*-value of E−05 [45]. We used the MAFFT server for multiple sequence alignment and MEGA to extract the amino acid at the edited site [46, 47]. Importantly, we excluded partial sequences, missing data, or sequences of uncultured/unclassified species, as well as unnamed or hypothetical proteins (Supplementary Tables S9 and S10).

**Figure 2.**
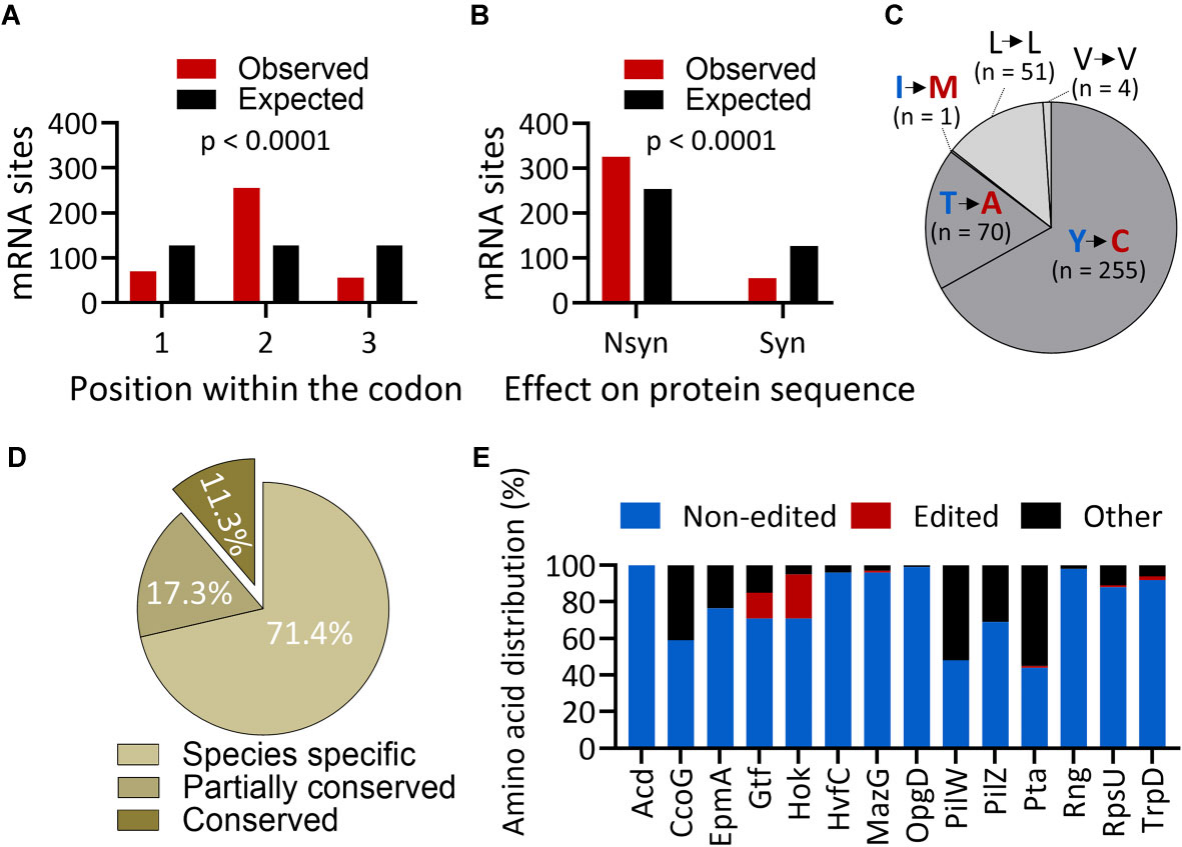
Hundreds of mRNAs are edited and predicted to recode protein sequences across 64 gammaproteobacterial species. (**A**) Distribution of observed (red) and expected (black) positions of editing events within codons (*P*-value <.0001; chi-square test for goodness of fit; marked in red). (**B**) Distribution of observed (red) and expected (black) effects of editing events on protein sequences (*P*-value <.0001; chi-square test for goodness of fit; marked in red). (**C**) Distribution of observed effect of editing events on protein sequences. (**D**) The conservation of editing events. (**E**) Amino acid identity at the position recoded by conserved editing events across hundreds to thousands of gammaproteobacterial species.

### Bacterial strains, DNA and RNA extractions, and cDNA synthesis

*Acinetobacter baylyi* ADP1 was grown on LB medium (10 g tryptone, 10 g sodium chloride, and 5 g yeast extract per one liter) at 37°C; *Pseudomonas chlororaphis*(ATCC 17415) on nutrient broth (Difco, #234000) at 26°C; *Vibrio alginolyticus* (ATCC 17749) on marine broth (Millipore, #76448) at 37°C; and *E. coli* MG1655-EcM2.1 on LB at 34°C. At mid-log (OD of 0.5–1) or stationary phase (24 h of growth, only *E. coli*), 1.5 ml was taken for DNA and RNA extractions. DNA was extracted using a GeneJET Genomic DNA Purification Kit (Thermo Scientific, #K0721), while RNA was extracted using a GeneJET RNA Purification Kit (Thermo Scientific, #K0731). RNA samples were treated with four units of DNase I (NEB, #M0303L) for 20 min at 37°C. Finally, following incubation of 15 min at 65°C, cDNA synthesis was done using GoScript Reverse Transcription Mix (Promega, #A2801). To synthesize cDNA, 500 ng of total RNA were primed with random hexamers and reverse-transcribed with the GoScript Reverse Transcription Mix Kit (Promega, #A2801) following the manufacturer’s protocol.

### RNA editing motif analysis

Using CLC Genomics Workbench, we extracted the four-base sequences surrounding all 1751 A-to-G mismatches. To exclude that our results stem from genomic bias—having, for some reason, enrichment of the TACG motif in the genome of the examined species—we extracted all four-base combinations for each species with adenosine at the second position. The frequencies of each four-base motif were calculated and compared to the observed motif frequencies using a chi-square test for goodness of fit.

The seven-base motif (YTACGAA) was identified using WebLogo analysis [48]. In short, we extracted the 21 bases surrounding each mRNA edited site (10 bases upstream and downstream). These 381 sequences were analyzed in WebLogo Server under default parameters (Supplementary Tables S3 and S11).

**Figure 3.**
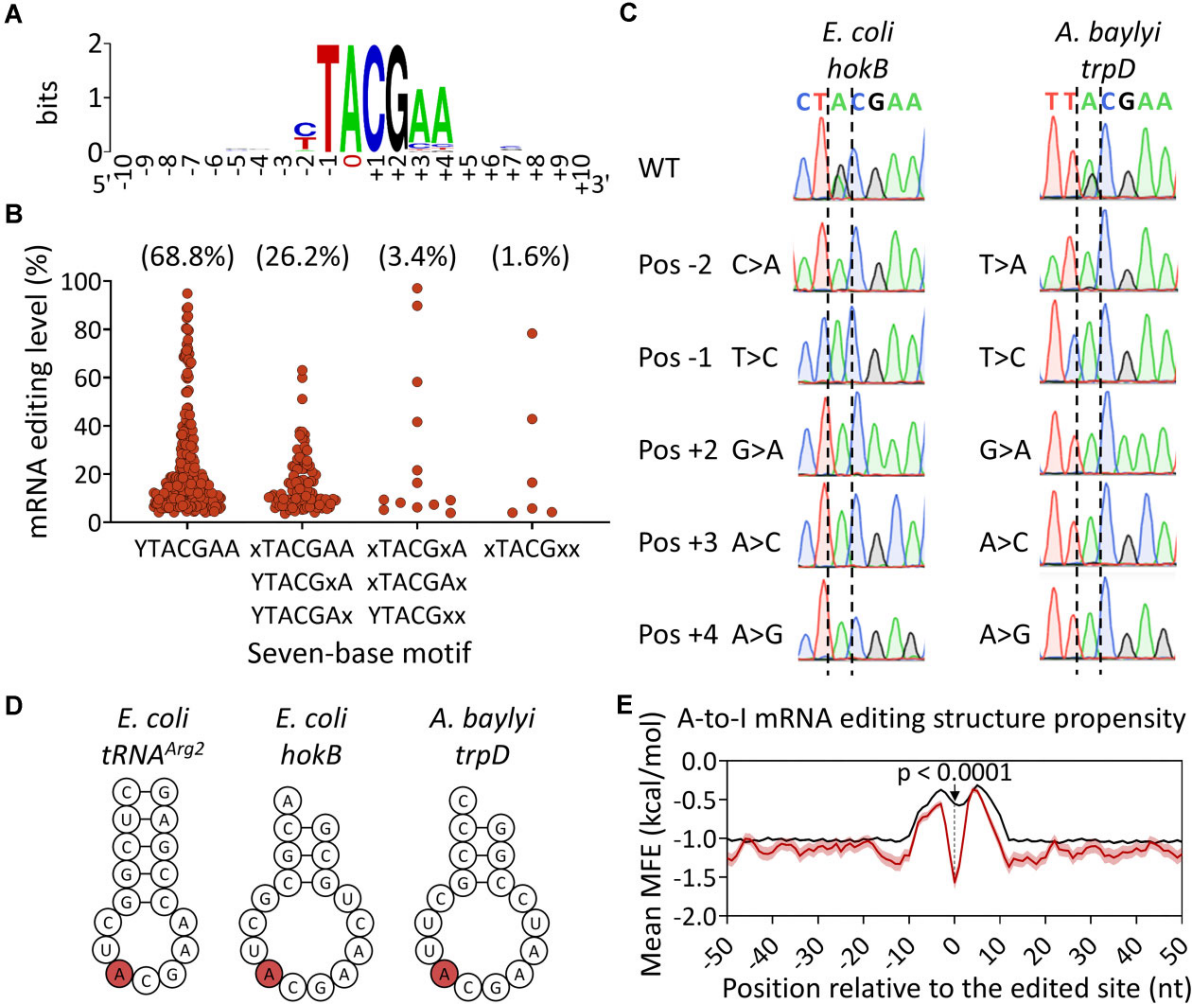
Bacterial mRNA editing occurs in a conserved seven-base motif embedded within a stem-loop structure. (**A**) WebLogo [48] of the 381 mRNA editing events detected in 64 gammaproteobacterial species. Position “0” is the edited site. (**B**) Distribution and editing levels of the 381 mRNA editing events across different seven-base motif combinations (data are provided in Supplementary Table S11). Mismatches to the conserved seven-base motif are marked in “x”. (**C**) Individually mutating different positions in the motif completely or nearly completely abolishes editing in two transcripts from different species. (**D**) Minimum free energy (MFE) secondary structure predicted by RNAfold [49] around the A-to-I editing site (red) for the 17 nucleotides composing the anticodon arm of *tRNA^Arg2^*, and for the 17 nucleotides around the edited site in the transcripts of *hokB* and *trpD*. (**E**) MFE (mean and standard error) predictions for 17-nucleotide sliding windows centered on the positions indicated relative to all A-to-I mRNA editing events (*n* = 381) and control sites harboring all other YTACGAA motifs from all examined species (42 813 sites from 64 species). Statistical analysis on the 17 nucleotides surrounding A-to-I mRNA editing events and control sites (at position “0”) was conducted using Welch’s *t*-test (marked with a black arrow).

### Secondary structure prediction around editing events

We used RNAfold from the ViennaRNA Package 2.0 [49] to calculate the MFE around editing events. As previously shown on editing events in *S. pyogenes* [42], we used a sliding window approach around each editing event with a window size of 17 nucleotides (which is the length of *tRNA^Arg2^* anticodon arm). We position the edited adenosine as the “0” position in our sliding window analysis similarly to its location in the anticodon arm of *tRNA^Arg2^* (NNNNNYT[A]CGAANNNNN). MFE average and standard error were calculated over all sliding windows’ positions for all editing events. Control positions containing the YTACGG motif were retrieved from the genomes of the 64 species with putative mRNA editing events. We used the “locate” command from the “SeqKit” program to identify all possible motifs’ locations in each genome and an R script to extract the genomic sequences flanking the motif site while filtering the real editing sites.

### Mutating genes in *E. coli* and *A. baylyi*

To mutate the motif around *hokB* in *E. coli*, we used *E. coli* strain MG1655-EcM2.1 (a specially designed strain for high MAGE efficiency) to carry out one MAGE cycle as previously described [50, 51]. We used five 90-base single-strand oligonucleotides with two phosphorothioate between the last three bases of the 5′ and 3′ ends to target the lagging strand in the *hokB* gene (Supplementary Table S12). Briefly, cells were grown overnight at 34°C. Then, 30 μl of the saturated culture was transferred into fresh 3 ml of LB medium in a 12-ml tube until reaching OD = 0.5 (measured in 1-cm cuvette in this section) and then moved to a shaking incubator in an Erlenmeyer containing 200 ml of water (200 RPM) at 42°C for 15 min after which it was moved immediately to ice. Next, 1 ml was transferred to an Eppendorf tube, and cells were washed twice with ice-cold double distilled water (DDW) at a centrifuge speed of 13 000 × *g* for 30 s. Next, the bacterial pellet was dissolved in 50 μl of DDW containing 2 μM of single-stranded DNA oligo and transferred into a cuvette. Electroporation was performed at 1.78 kV, 200 Ω, and 25 μF. After electroporation, the bacteria were transferred into 3 ml of fresh LB and incubated at 34°C until reaching OD = 0.8. Then, they were diluted in a 1:10^−3^ ratio followed by plating 25 μl on LB-agar-ampicillin plates (100 μg/ml). To identify positive MAGE colonies (referred to as bacterial strains throughout the text), we PCR (polymerase chain reaction)-amplified fragments encompassing the *E. coli* genomic region with primers corresponding to the mutated and WT form (differing in one base in their 3′ ends). Successful/strong PCR amplification implies successful MAGE mutagenesis.

*Acinetobacter baylyi* ADP1 mutants were created using the Golden Transformation method described in [52]. Briefly, DNA fragments homologous to 1 kb upstream and downstream of each target site were amplified by PCR (Phusion High-Fidelity PCR Kit, New England Biolabs) with primers containing additional Golden Gate ligation adaptors (Supplementary Table S12). These flanks were then ligated to a *tdk*-*kanR* dual selection cassette from plasmid pBTK622 using BsaI Golden Gate assembly (digested by BsaI-HFv2 and ligated by T7 DNA ligase, New England Biolabs). This product was transformed into ADP1 by incubating 35 μl of an overnight culture with 20 μl of ligation reaction in 500 μl of LB media. After overnight growth at 30°C, the culture was diluted 100-fold in sterile saline, and 50 μl was plated on LB plates containing kanamycin at 50 μg/ml. For the *trpD* mutants, the *tdk*-*kanR* cassette was inserted into the ADP1 genome, replacing the *trpD* gene. A rescue cassette was synthesized by ligating two PCR products containing the *trpD* coding sequences upstream and downstream of the targeted mutations, plus an additional 1 kb of homology on each end. The four-base overhangs used to ligate these two products together incorporated the single base pair substitution for each mutant. Each rescue cassette was transformed into the *trpD*::*tdk*-*kanR* strain and selected for transformants that replaced the *tdk* gene with the mutated *trpD* sequence on LB plates containing 200 μg/ml azidothymidine. To create the *tadA* mutant encoding TadA^D54E^, the *tdk*-*kanR* cassette was inserted three base pairs downstream of the *tadA* gene instead because attempts to insert the cassette within the *tadA* gene were unsuccessful. This strain was then transformed with a rescue cassette encoding the mutation, as for the *trpD* mutants. The homology between the mutated position in *E. coli* and *A. baylyi* was determined by multiple sequence alignment with web-based NCBI’s BLAST (Supplementary Fig. S3) [45].

**Figure 4.**
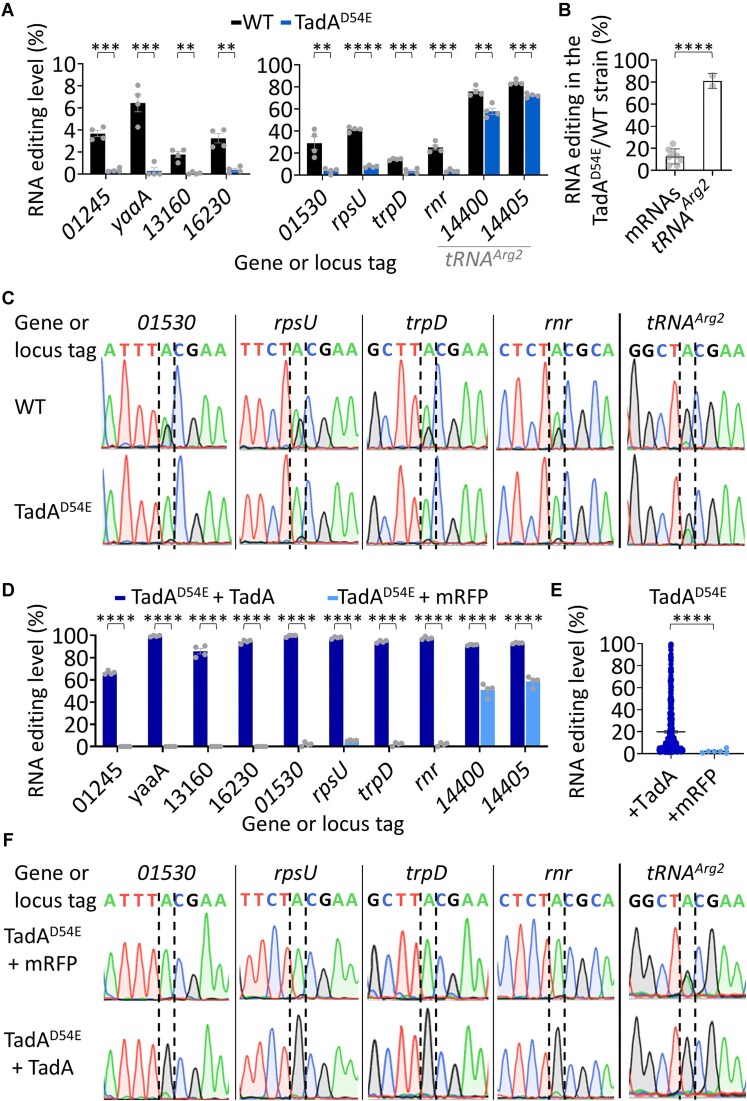
TadA mediates A-to-I mRNA editing in *A. baylyi* and has the potential to reshape the transcriptome. (**A**) RNA editing level (%) of mRNAs and *tRNA^Arg2^* in the WT and TadA^D54E^ mutant strains of *A. baylyi* determined from RNA-seq data. The means and standard errors of four biological replicates conducted on different days are shown (*N*= 4). (**B**) Relative change in editing levels in the TadA^D54E^ strain compared to the WT strain as measured by RNA-seq. Each dot represents the reduction in average editing level in each edited RNA. See calculations in Supplementary Table S13. (**C**) Sanger sequencing of the mRNAs and *tRNA^Arg2^* with the highest level of editing shown in panel (A). (**D**) RNA editing level (%) of mRNAs and *tRNA^Arg2^* in the TadA^D54E^ mutant supplemented with TadA^WT^ or mRFP from the pBTK402 plasmid determined from RNA-seq data. The means and standard errors of four biological replicates conducted on different days are shown (*N* = 4). (**E**) Average and standard error of mRNA editing events detected in the TadA^D54E^ strain overexpressing TadA^WT^ (433 sites) or mRFP (6 sites) from the pBTK402 plasmid. Each dot represents the average editing level of a given site as detected in all four biological replicates. (**F**) Sanger sequencing of the mRNAs and *tRNA^Arg2^* with the highest level of editing shown in panel (C). Statistical analysis in panel (A), (B), and (D) was conducted using Student’s *t*-test followed by Benjamini–Hochberg false discovery rate (FDR) correction (in panels A and D), and in panel (E) using Welch’s *t*-test: *P*-value ≤.01 (**), ≤.001 (***), and ≤.0001 (****).

To verify successful mutagenesis of all genes, we amplified a fragment encompassing the mutated position, and Sanger sequenced it. Primers for all PCR reactions are shown in Supplementary Table S12. The sequences were aligned and visualized using SnapGene (Dotmatics).

### Plasmid construction for TadA overexpression

In order to express TadA from a plasmid, we used the pBTK402 plasmid backbone (Addgene plasmid, #110598) [53]. First, we linearized the pBTK402 backbone without the *mrfp* gene. In addition, we PCR amplified the *tadA* gene from *A. baylyi* genome with 18-base overlap to the pBTK402 linearized PCR product. We then used NEBuilder^®^ HiFi DNA Assembly to replace the *mrfp* gene with *tadA* and cloned it into *E. coli* (DH10B). The plasmid was extracted using ZymoPURE Plasmid MiniPrep Kit (#ZR-D4209), sequenced to validate successful construction, transformed into *A. baylyi* as described above when mutating its genome, and plated on LB–kanamycin plates. PCR was conducted using the primers listed in Supplementary Table S12.

### RNA-seq and RNA editing analysis of *A. baylyi* WT and TadA^D54E^ strains

Samples were grown in LB medium, each started from a single and different colony. RNA was extracted in mid-log phase at OD of 0.5–0.8 as described above. Ribosomal RNA was depleted using NEBNext^®^ rRNA Depletion Kit (Bacteria) (New England Biolabs, #E7850). Libraries were constructed using NEBNext^®^ Ultra™ II Directional RNA Library Prep Kit for Illumina^®^ (New England Biolabs, #E7760). Finally, RNA-seq libraries were sequenced on the NovaSeq X platform (Illumina).

RNA editing analysis was performed as described above with minor modifications. First, the editing frequency was set to 1%. Second, for an editing event to be identified, we required it to be found in at least three out of four biological replicates of the WT strain. Third, we excluded DNA variants identified in a DNA-seq sample of *A. baylyi*. Fourth, as our RNA-seq was strand-specific, we could filter for “true” transcript-to-reference genome A>G mismatches. Fifth, to make sure editing events were not missed due to our stringent parameters (e.g. because they lack at least 10 reads covering a site), we extracted the read coverage and variant data from the sequence mappings in samples where the variant was not identified (Supplementary Tables S13–S15).

### Growth assays

*Acinetobacter baylyi* cultures were grown at 37°C for 24 h in LB medium, back diluted in a 1:100 ratio to a 50-ml conical tube containing 10 ml LB medium, vortexed, and dispensed (150 μl) to 96-well plates (Corning Costar). Wells were measured every 30 min for optical density at OD_600_ for 24 h (Synergy H1, Biotek). The 96-well plate was divided as follows: 12 wells were blank control (line A of the plate), and the remaining 84 wells were divided between the bacterial stains. For each strain, growth curves were obtained for technical replicates (wells in the plate), as indicated in each figure. Two identical plates were created from the same starter and grown separately (in parallel) in two different Synergy H1 plate readers at 37°C and 42°C. We conducted five independent experiments (biological repeats), on different days with starters from different colonies.

### Proteolysis

From starter cultures grown for 24 h, *A. baylyi* WT and TadA^D54E^ strains were grown at 37°C in LB (3–7 h) or MS media (7–16 h) to an OD_600_ of 0.46–0.78. Bacteria were centrifuged (Eppendorf 5425R) at 13 000 × *g* for 30 s and washed with 1× PBS three times and stored at −20°C. Bacterial cells were lysed in 8.5 M urea, 400 mM ammonium bicarbonate, and 10 mM DTT, sonicated twice (90%, 10–10, 5′). Protein amounts were estimated using Bradford readings. We loaded 20 μg of proteins from strains 107 and 118 (grown on LB) on 4%–15% gradient sodium dodecyl sulfate–polyacrylamide gel electrophoresis in three replicates. We cut out three slices from each line in different molecular weights from the gel: 92 kDa for Rnr, 37 kDa for TrpD, and 8 kDa for RpsU. The proteins in the gel slices were reduced with 3 mM DTT (60°C for 30 min), modified with 10 mM iodoacetamide in 100 mM ammonium bicarbonate (in the dark, room temperature for 30 min), and digested in 10% acetonitrile and 10 mM ammonium bicarbonate with modified trypsin (Promega) at a 1:10 enzyme-to-substrate ratio, overnight at 37°C. An additional second digestion with trypsin was done for 4 h at 37°C. The tryptic peptides were desalted using HLB μElution plate (Waters) and re-suspended in 0.1% formic acid.

### Mass spectrometry analysis

The resulting peptides were analyzed by Liquid Chromatography coupled with Tandem Mass Spectrometry (LC–MS/MS) using an Exploris 480 mass spectrometer (Thermo) fitted with a capillary HPLC (EVOSEP ONE, Evosep).

The peptides were loaded onto a 15-cm, ID 150-μm, 1.9-μm Endurancse column EV1137 (Evosep). The peptides were eluted with the built-in 15 SPD (88 min) method.

Mass spectrometry was performed in a positive mode using repetitively full MS scan (*m/z* 350–1200) followed by high-energy collision dissociation in two separate scan events. First event: the 20 most dominant ions (>1 charge) were selected from the full MS scan; a dynamic exclusion list was enabled with an exclusion duration of 30 s. A second scan event: the 20 most dominant ions (>1 charge) were selected from a mass list without dynamic exclusion.

### Mass spectrometry data analysis

Mass spectrometry data were analyzed using Protein Discoverer 2.4 (Thermo) using the Sequest search engine, searching against the *A. baylyi* proteome (UP000000430) from the UniProt database (downloaded on 9/11/2023, 3263 entries) and specific sequences of edited proteins (with the different amino acids), with a mass tolerance of 20 ppm for the precursor masses and 0.02 Da for the fragment ions. Oxidation of methionine and N-terminal acetylation were accepted as variable modifications, and carbamidomethyl modification of cysteine was accepted as a static modification. The minimal peptide length was set to six amino acids, and a maximum of two miscleavages were allowed. Data were quantified by label-free analysis using the same software. Peptide-level FDRs were filtered to 1% using the target-decoy strategy.

### Statistical analysis

We used PRISM 10 to conduct the statistical analyses described in the text, except for peptide-level FDRs calculated for the mass spectrometry analysis.

## Results

### A-to-I mRNA editing is widespread in gammaproteobacteria

To determine the prevalence of A-to-I mRNA editing across different species, we analyzed 554 published RNA-seq experiments of gammaproteobacterial species (Supplementary Table S1), each with at least two biological replicates (Supplementary Table 2). We focused on the class of gammaproteobacteria because editing was first discovered in *E. coli*, supporting that editing might be found in additional members of this class. Furthermore, many human pathogens belong to this class, increasing the importance of understating the prevalence of editing in these species.

We used stringent parameters (see the “Materials and methods” section and Supplementary Figs S1 and S2A and B) and identified 1751 A-to-G mismatches in 72 bacterial species, which could represent RNA editing events, DNA mismatches (mutations) between the sequenced strain to the reference genome, or technical noise (Supplementary Table S3). Among the 1751 A-to-G mismatches, 571 (32.6%) occur in a TACG motif, which is the motif TadA requires for RNA editing activity in *E. coli* (Fig. 1A–C and Supplementary Table S3). This enrichment in the TACG motif is statistically significant (χ^2^_(df = 63, *N* = 1751)_ = 16864, *P*-value <.0001) and supports a TadA-dependent editing mechanism for these cases (Fig. 1C). Furthermore, TadA is encoded by the genome of all species with identified putative editing events in the TACG motif (Supplementary Table S7). Therefore, we focused on the 571 TACG-embedded putative sites, found in 64/72 species (88.8% of species) for downstream analysis.

Analyzing the 571 putative RNA edits revealed that 381 occur within mRNAs, 187 in *tRNA^Arg2^* (encoded by multiple genes of *tRNA^Arg2^* per species), and 3 in the 23S rRNA (Supplementary Fig. S2C and Supplementary Table S4).

To validate that our analysis detected true mRNA editing events, we used Sanger sequencing to analyze matched DNA–RNA samples from three representative species—*A. baylyi*, *P. chlororaphis*, and *V. alginolyticus*. We focused on sites with an average predicted editing level of 20% and above in the RNA-seq datasets (Fig. 1D and Supplementary Table S4), so they would be detectable in Sanger sequencing traces. We validated 10/11 sites (90.1%) across the three species (Fig. 1E and Supplementary Fig. S4). The one site that was not detected in Sanger could represent a false positive site, or it is a real site that, in our strain or growth conditions, is not edited.

Combined, our results support that *bona fide* A-to-I mRNA editing is widespread in gammaproteobacterial species and is predicted to change protein sequences in most cases.

### A-to-I mRNA editing is expected to alter protein sequences in most instances and can be conserved across different bacterial species

Given the ability of inosine to be recognized as guanosine by the ribosome, we examine the predicted effect of editing on bacterial protein sequences. Most A-to-I mRNA editing events (85.5%; *n* = 326) are predicted to recode protein sequences with a frequency significantly higher than expected at random, supporting that non-synonymous editing events might be positively selected in bacteria (Fig. 2A). Furthermore, most editing events are found at the second position of the codon, in a significantly higher frequency than expected by random sampling (Fig. 2B). Conversely, editing events found at the codon’s first and third positions are significantly less represented than expected. Because mRNA editing sites are embedded in the UACG motif, they almost exclusively result in threonine to alanine (T→A) or tyrosine to cysteine (Y→C) substitutions when they recode the first position in ACG→ICG (GCG) and the second position in UAC→UIC (UGC), respectively. Consequently, among the non-synonymous editing events, 255 changed a tyrosine to a cysteine codon, 70 changed a threonine to an alanine codon, and 1 changed an isoleucine to a methionine codon (Fig. 2C and Supplementary Table S5). We also detected 55 synonymous editing events, altering the third position of lysine codons (UUA and CUA) in 51 events, and that of a valine codon (GUA) in 4 events (Fig. 2C and Supplementary Table S6). Thus, A-to-I mRNA editing might constitute a novel advantageous mechanism, especially for introducing cysteines into bacterial proteins, with phenotypic consequences that are yet to be discovered.

Next, we aimed to examine to what degree editing events could be conserved. Among the 381 A-to-I editing events, most are unique (*n* = 272), which means that they are found in mRNAs that encode different proteins in different species (Fig. 2D). We also observed editing events that are partially conserved (*n* = 66), which means that they are found in transcripts that are predicted to encode the same protein or protein family in at least two different species, but do not recode the same position or amino acid in the translated protein (Fig. 2D and Supplementary Table S8). The remaining set of the editing events (*n* = 43) are completely conserved between species belonging to the same genus, family, order, and/or class, and recode the same amino acid at the same position in the predicted translated protein (Fig. 2D and Supplementary Table S8).

Finally, we focused on the conserved editing events and examined the identity of the amino acid at the edited site in thousands of gammaproteobacterial protein homologs. Generally speaking, the non-edited amino acid was the most prevalent (Fig. 2E and Supplementary Tables S9 and S10). In contrast, the edited/recoded amino acid was in low frequency or completely absent across the examined protein homologs (Fig. 2E). Importantly, the analyzed protein sequences are predicted from the DNA that encodes them. Thus, having the edited codon “hard-coded” at the DNA level is selected against for some reason.

In summary, A-to-I mRNA editing is predicted to affect protein sequences and introduce amino acids not encoded at the DNA level across gammaproteobacteria.

### Bacterial A-to-I mRNA editing requires an evolutionarily conserved seven-base motif embedded within a stem-loop structure

Next, we aimed to understand the principles governing mRNA editing in the examined bacterial species. As mentioned above, TadA-dependent tRNA and mRNA editing requires a four-base motif around the edited adenosine, both *in vivo* and *in vitro* [35, 36, 42]. However, the motif required for editing is possibly longer, given the seven bases of conservation around editing events in both mRNAs and *tRNA^Arg2^* of *E. coli* and *S. pyogenes* [35, 42]. Now, with hundreds of mRNA editing events from dozens of species at our disposal, we examined the level of sequence conservation around editing events from an evolutionary perspective. Using WebLogo [48], we analyzed a 21-base sequence (±10 bases) around the 381 mRNA editing events and discovered sequence conservation of seven bases (YTACGAA), which matches the anticodon loop sequence and length of *tRNA^Arg2^* (Fig. 3A). While 68.8% of editing events were embedded in the YTACGAA motif, 26.2% occurred in a sequence motif that differs by one base (Fig. 3B). Larger deviations, of two or three nucleotides, from the consensus motif occurred in only 5% of the sites (Fig. 3B).

Next, we experimentally examined the functional importance of different positions in the motif for mRNA editing formation. Previously, we showed that mutating the downstream nucleotide that is adjacent to the edited site (position +1; C>T) within the canonical TACG motif completely abolished mRNA editing in the transcript of *hokB* in *E. coli* [35]. Thus, we used the same strategy to individually mutate the remaining positions of the motif in the transcript of *hokB* in *E. coli* and the transcript of *trpD* in *A. baylyi*. Mutating these five sites completely or nearly completely abolished mRNA editing in both cases (Fig. 3C).

Finally, we examined whether our identified editing events occur within a stem-loop structure as reported for *tRNA^Arg2^* editing and mRNA editing in *E. coli* and *S. pyogenes* [35, 36, 42]. Indeed, the MFE was significantly lower (*P*-value ≤.0001) around editing events compared with the control sites, supporting the presence of a secondary structure (Fig. 3D and E). Thus, our results suggest the existence of negative selection against the formation of an RNA secondary structure around YTACGAA containing sites with no editing. We hypothesize that this negative selection prevents unwanted editing across the bacterial transcriptome.

In conclusion, our computational and experimental data support that TadA-dependent A-to-I mRNA editing occurs and requires a conserved seven-base motif embedded within a stem-loop structure.

### TadA mediates A-to-I mRNA editing in *A. baylyi* and has the potential to reshape the transcriptome

To further examine that our analysis detected *bona fide* TadA-dependent A-to-I mRNA editing events, we focused on *A. baylyi*, a soil bacterium with diverse molecular tools for genetic engineering [54]. Moreover, unlike *E. coli*, *A. baylyi* exhibits high editing levels in multiple mRNAs, making it an ideal bacterial model to investigate the importance of A-to-I mRNA editing in bacteria. Thus, we created a strain of *A. baylyi* encoding a mutant version of TadA (D54E) at its native chromosomal locus. An equivalent substitution was shown to reduce mRNA editing levels *in vivo* and *in vitro* in *E. coli* [35, 36].

To examine the effect of the D54E substitution in *A. baylyi*, we used RNA-seq to detect RNA editing events in the WT and TadA^D54E^ strains. We identified 11 A>G RNA-specific mismatches to the reference genome. Among these 11 A>G mismatches, the variant frequency (putative editing level) of 10 was significantly reduced in the TadA^D54E^ mutant compared to the WT strain, across all samples (Fig. 4A and Supplementary Table S13). Among these 10 A>G mismatches, 9 were embedded in an exact match to the YTACGAA motif and 1 was in a single base mismatch to this motif (Fig. 4A and Supplementary Table S13). In total, we identified eight editing events in mRNAs and two events in the anticodon of *tRNA^Arg2^* transcribed from two *tRNA^Arg2^* genes (Supplementary Table S13). Notably, in the TadA^D54E^ mutant compared to the WT strain, editing levels were significantly more reduced in mRNAs than in *tRNA^Arg2^* (Fig. 4A and B, and Supplementary Table S13). Sanger sequencing validated the reduction in editing level in four mRNAs and *tRNA^Arg2^* with the highest level of editing (Fig. 4C).

To further investigate the involvement of TadA in A-to-I mRNA editing, we overexpressed either TadA or mRFP (control) from a plasmid in the TadA^D54E^ mutant and performed RNA-seq. We observed a significant increase in editing levels in all mRNA and *tRNA^Arg2^* sites (Fig. 4D and Supplementary Table S14). Moreover, upon TadA overexpression, we detected a total of 433 mRNA editing events (Fig. 4E and Supplementary Table S15). While most sites (69.1%) reside within the canonical four-base motif (TACG), the rest reside in a sequence that deviates by one (29.3%) or two (1.6%) bases from this motif (Supplementary Fig. S5A and Supplementary Table S16). Similarly, most sites (72%) match the YTACGAA motif or are one base substitution away from it (Supplementary Fig. S5B and Supplementary Table S17). Finally, Sanger sequencing validated the increase in editing level in four mRNAs and *tRNA^Arg2^* (Fig. 4F).

The conservation of the editing motif across 64 bacterial species, combined with our experimental results in *A. baylyi* and previous experimental work in *E. coli* [35] and *S. pyogenes* [42], supports that TadA mediates both mRNA and tRNA editing across bacteria. Furthermore, our work suggests that elevated levels of TadA can increase editing levels and the number of edited mRNAs, thus reshaping the bacterial transcriptome.

### A-to-I mRNA editing enables bacteria to produce two protein versions from a single gene

**Figure 5.**
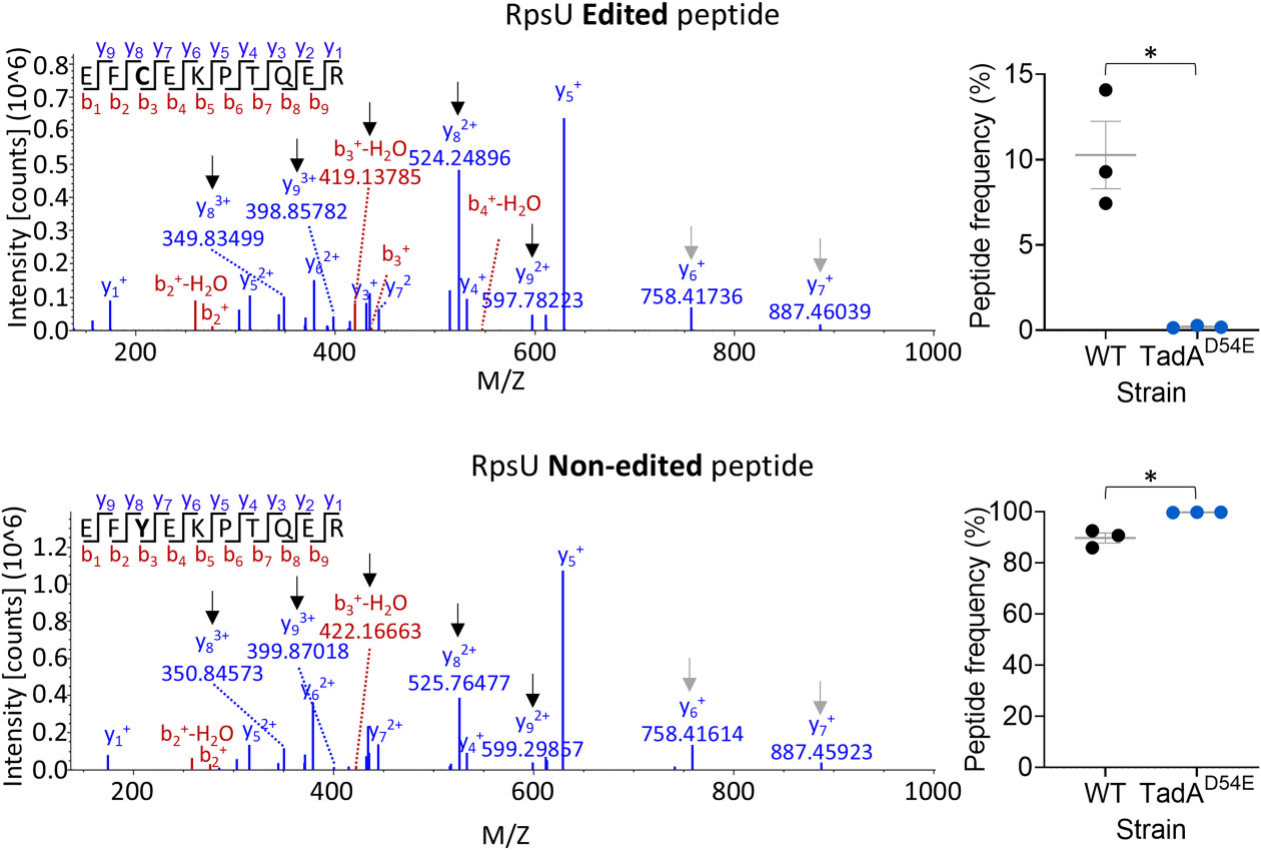
A-to-I mRNA editing introduces protein isoforms in bacteria. Left: Representative MS/MS spectrum of edited (C38; top) and non-edited (Y38; bottom) RpsU peptides, and their normalized frequencies. Black arrows mark identified peptides and their mass in the MS/MS spectra that show a shift in mass corresponding to the presence of a tyrosine or cysteine at the edited site and can be compared between the two MS/MS spectra (different mass). The gray arrow marks an example of a peptide and its mass in the MS/MS spectra that does not include the edited site (same mass). All peptides were discovered with FDR ≤ 0.01. The peaks’ weight, font size, and axis were adjusted from the original figure for better visualization and only the peaks of identified peptides that correspond to the edited or non-edited peptides are shown. A comprehensive mass distribution and the original MS/MS spectra can be found in Supplementary Fig. S6 and Supplementary Table S18. Right: Relative peptide frequencies in the TadA^D54E^ mutant and the WT strain of *A. baylyi*. The mean and standard error of three biological replicates conducted on different days (*N* = 3) are shown. Statistical analysis was conducted using Welch’s *t*-tests: *P-value ≤.05.

Bacteria are haploid organisms. Thus, protein recoding by A-to-I mRNA editing could constitute a novel mechanism to diversify their proteome. To test this possibility, we performed targeted mass spectrometry on *A. baylyi* WT and TadA^D54E^ strains, focusing on RpsU, Rnr, and TrpD (Fig. 4A and B). We chose these proteins as their transcripts harbored the highest editing level in *A. baylyi* (average editing level >14%) that is also predicted to result in an amino acid substitution (Fig. 4 and Supplementary Table S13). In RpsU, we detected both the edited and non-edited peptides in all three biological replicates of the WT strain (Fig. 5, Supplementary Fig. S6, and Supplementary Table S18). Moreover, the frequency of the edited peptide of RpsU significantly decreased in the TadA^D54E^ strain compared to the WT (Fig. 5, Supplementary Fig. S6, and Supplementary Table S18). Conversely, the frequency of the non-edited peptide of RpsU significantly increased in the TadA^D54E^ strain compared to the WT (Fig. 5, Supplementary Fig. S6, and Supplementary Table S18). We also discovered the edited peptide of Rnr in one out of three biological replicates of the WT strain, but not in any of the TadA^D54E^ replicates (Supplementary Fig. S6 and Supplementary Table S18). In contrast, we only discovered the non-edited peptide of TrpD (Supplementary Fig. S6 and Supplementary Table S18). Our lack of ability to detect edited TrpD can stem from technical reasons such as lower editing levels (compare to RpsU and Rnr) that result in undetectable levels of the edited peptide. Alternatively, it is possible that the edited peptide of TrpD is not translated or is unstable for some reason. In conclusion, A-to-I mRNA editing enables bacteria to produce two protein versions from a single gene as demonstrated in the cases of RpsU and Rnr.

### TadA mutant with deficient editing activity does not grow at high temperatures

**Figure 6.**
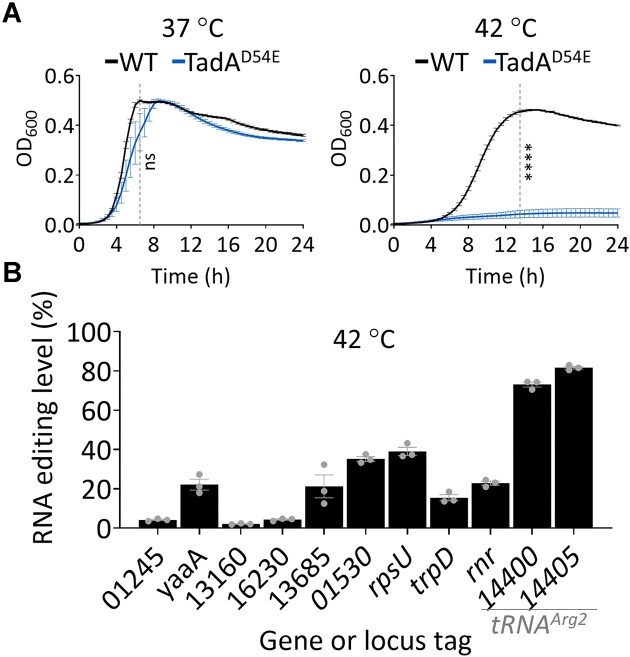
TadA mutant with deficient editing activity does not grow at high temperatures. (**A**) Growth assays with the WT and TadA^D54E^ mutant strain of *A. baylyi* in LB. The mean and standard error of five biological replicates conducted on different days (*N* = 5), each with 42 technical replicates, are shown. The dashed gray line represents the end of the log phase in the WT strain and was used for statistical analysis using Student’s *t*-tests: ****P-value ≤.0001. (**B**) A-to-I RNA editing level (%) of mRNAs and *tRNA^Arg2^* in WT *A. baylyi* determined from RNA-seq data of samples that grew at 42°C to mid-logarithmic phase. The means and standard errors of three biological replicates conducted on different days are shown (*N* = 3).

Next, we aimed to test the functional effect of having a mutated TadA with reduced activity in *A. baylyi*. Previously, A-to-I mRNA editing was suggested to have a temperature-dependent role in flies [7, 55, 56], nematodes [57], octopuses [58], and zebrafish [59]. Therefore, we compared the growth of the WT and TadA^D54E^ strains at different temperatures, 37°C and 42°C. We observed that at 37°C, the TadA^D54E^ strain did not display a significant growth defect compared to the WT (Fig. 6). Strikingly, at 42°C, the TadA^D54E^ strain did not grow, even after 24 h (Fig. 6A). Moreover, RNA-seq revealed that RNA editing in both tRNA and mRNA editing activity occurs endogenously at 42°C (Fig. 6B). Thus, tRNA or mRNA A-to-I editing (or both) possibly contributes to bacterial growth at 42°C.

The growth defect observed in the TadA^D54E^ mutant could stem from decreased mRNA or *tRNA^Arg2^* editing levels (or both). To decouple the effect of mRNA and tRNA editing, we created mutants encoding at their native chromosomal locus only the non-edited or edited version of three protein-coding genes (*rpsU*,*trpD*, and *rnr*). To create the non-edited version, we mutated the motif around the edited site to obtain a synonymous mutation. Thus, the translated protein is expected to solely have the same amino acid as encoded by the DNA sequence (Supplementary Fig. S7A). Unlike in the TadA^D54E^ strain, where we observed a reduction in mRNA editing levels, in the non-edited strain, we observed a complete loss of editing (Supplementary Fig. S7A). To create the edited-only strain, we mutated the edited adenosine to guanosine at the DNA level, introducing a codon for the amino acid that would be translated from an edited mRNA (Supplementary Fig. S7A). In all tested cases, we did not observe decreased fitness in the mutant strains at 37°C or 42°C (Supplementary Fig. S7B). Thus, our experiments suggest that *A. baylyi* can withstand perturbation of editing in a single gene under laboratory conditions. However, this does not exclude that all or most mRNA editing events have a cumulative functional role or that individual editing events serve a purpose under different environmental conditions.

## Discussion

Bacteria are haploid organisms with a single copy of each gene in their genome. Here, we show that A-to-I mRNA editing occurs in dozens of bacterial species, enabling bacteria, as demonstrated by the case of *A. baylyi*, to produce two protein versions from a single gene (Figs 1, 4, and 5). However, the intrinsic and extrinsic factors that affect the occurrence of mRNA editing in bacteria and the consequences of these editing events on protein function and organismal fitness are poorly understood.

We have shown that several intrinsic factors impact levels of A-to-I mRNA editing. Our analysis of editing events across gammaproteobacteria shows that a seven-base motif is required for TadA editing (Fig. 3 and Supplementary Table S11). Editing events in these species are favored when this motif occurs in an RNA stem-loop structure. The importance of this structural context has been established in *E. coli* and *S. pyogenes* through either mutagenesis studies or computational analysis [35, 36, 42].

Why does mutant TadA edit *tRNA^Arg2^* to a greater extent than the mRNA targets? It is possible that differences in their structural and genomic contexts are responsible. Compared to *tRNA^Arg2^*, the mRNA targets may not fold as stably or as reliably into the stem-loop structure required for TadA editing. We also expect that *tRNA^Arg2^* is more stable to nuclease degradation than mRNAs, which would provide more time for the mutant TadA with reduced activity to edit its adenosine. Whether an RNA target is being actively translated is also likely to affect how efficiently it is edited. TadA will have to compete with ribosomes for access to editing sites in mRNAs, which is not the case for *tRNA^Arg2^*. Still, other alternatives exist, such as whether the spatial organization of the bacterial chromosome and genes within it affect the editing process. If edited transcripts are encoded near *tadA* on the chromosome or found close to *tadA* due to the 3D structure of the folded/condensed chromosome, it may increase the probability of TadA encountering and editing these RNAs. Future work should examine the above possibilities (and others) using gene localization replacements, microscopy, and translation assays.

Another intrinsic factor that directly affects editing efficiency at a given site is TadA expression. We showed that overexpressing TadA from a plasmid resulted in 433 mRNA editing events in 329 transcripts (Fig. 4 and Supplementary Table S15). We previously observed a similar increase in editing events and levels in *E. coli* upon overexpression of TadA [35]. In contrast, when TadA was overexpressed from a plasmid in *S. pyogenes*, only a handful of novel sites were edited [42]. This difference in editing response could stem from technical reasons, such as the expression vector used in *S. pyogenes* not increasing protein product by the same amount. Alternatively, the ability of TadA to edit additional sites as a function of its expression level might be different between Gram-negative (*A. baylyi* and *E. coli*) and Gram-positive bacteria (*S. pyogenes*).

Temperature is an important extrinsic factor that affects mRNA editing efficiency. Recent work by Wulff *et al.* showed that temperature can affect mRNA editing levels in different transcripts in *S. pyogenes* [42]. We found that TadA activity is required for the growth of *A. baylyi* at high temperatures (Fig. 6). As TadA edits both tRNAs and mRNAs, future work will be needed to decouple whether this phenotype stems from a loss of tRNA or mRNA editing. We did not observe a difference in growth under laboratory conditions when editing was blocked in individual mRNAs (Supplementary Fig. S7). It could be that the tested mRNA editing events do not contribute to the observed phenotype in the TadA mutant strain. Alternatively, it could be that mRNA editing events contribute collectively to the fitness of *A. baylyi* at 42°C (Fig. 6) or that these editing events are important for *A. baylyi* fitness in other environments.

The physiological roles that A-to-I mRNA editing events play in bacteria and their consequences on fitness are still largely unknown. Genome-wide, one would expect selection against the appearance of mRNA structures containing the TadA motif when editing would result in a loss of protein function that is deleterious to a cell. It is possible that all or most editing events observed in mRNAs are “accidental.” That is, they are off-target sites at which editing is effectively neutral with respect to fitness. In these instances, both protein sequences translated from the edited and unedited mRNAs may be equally functional, or a mixture of protein variants with different activities may be enough to fully support the cell’s needs. However, it is also possible that at least some editing events that allow bacteria to express two versions of a protein are beneficial to fitness. This could occur if the two protein variants have complementary properties, such as each one supporting growth and survival under different environmental conditions. A final possibility is that due to neutral drift in sequence space, some proteins have become dependent on an editing event and will not fold or function without it. In these cases, the editing event was not beneficial when it evolved, but it would now be deleterious to lose editing at this site.

Testing which of these circumstances explains the evolution of individual mRNA editing sites will require additional experimental work, such as mutating editing sites to lock in expression of one protein variant without disturbing gene expression *in vivo* or biochemically characterizing edited/unedited proteins variants *in vitro*. A further challenge in interpreting mRNA editing events is that their fitness effects may be condition-dependent and potentially masked when growing bacteria under laboratory conditions.

To conclude, our work revealed that A-to-I mRNA editing in bacteria is widespread and can reshape the bacterial transcriptome and proteome. Furthermore, our work sets the stage for future in-depth functional examination of the hundreds of mRNA editing events reported here across multiple bacterial species.

## Supplementary Material

gkaf656_Supplemental_Files

## Data Availability

Data will be available in a public, open-access repository upon publication. The mass spectrometry data were deposited to Pride through the ProteomeXchange in Project accession: PXD051887. The RNA-seq data were deposited to the NCBI SRA under accession PRJNA1106660.
