# Supplementary material for: A-to-I mRNA editing recodes hundreds of genes in dozens of species and produces endogenous protein isoforms in bacteria
Source: Nucleic Acids Res. 2025 Jul 12;53(13):gkaf656. doi: 10.1093/nar/gkaf656 (PMC12255301; doi:10.1093/nar/gkaf656)
Supplement: gkaf656_Supplemental_Files [file gkaf656_supplemental_files.zip › Elias_et_el_Supplementary_Data_20250528.pdf]

A-to-I mRNA editing recodes hundreds of genes in dozens of species and produces endogenous protein isoforms in bacteria

Eyal Elias[1], Isaac Gifford[2], Liron Didi[1], Ofir Fargeon[1], Danielle Arad[1], Rinat Cohen-Pavon[1], Gil Sorek[3], Liron Levin[4], Dganit Melamed[5], Liam Aspit[1], Jeffrey E. Barrick[2], and **Dan Bar-Yaacov**[1*]

[1]The Shraga Segal Department of Microbiology, Immunology, and Genetics, Ben-Gurion University of the Negev, Israel; [2]Department of Molecular Biosciences, The University of Texas at Austin, USA; [3]Department of Life Sciences, Ben-Gurion University of the Negev, Israel; [4]Bioinformatics Core Facility, llse Katz Institute for Nanoscale Science and Technology, Ben-Gurion University of the Negev, Beer-Sheva, Israel; [5]The Smoler Protein Research Center, Technion Israel Institute of Technology, Israel.

*Correspondence: danbary@bgu.ac.il

SUPPLEMENTARY FIGURES AND TABLES' legend

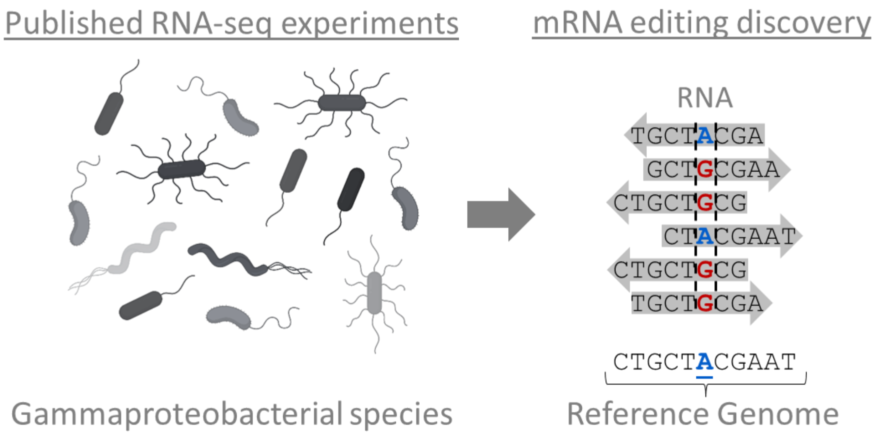

Supplementary Figure 1. Detecting A-to-I mRNA editing in gammaproteobacterial species. When RNA is sequenced from a bacterial sample by next-generation sequencing, if A-to-I RNA editing occurs in a specific site, the RNA reads covering that site will harbor both adenosine and guanosine (because the sequencing process identifies inosine as guanosine), while the reference genome will harbor adenosine.

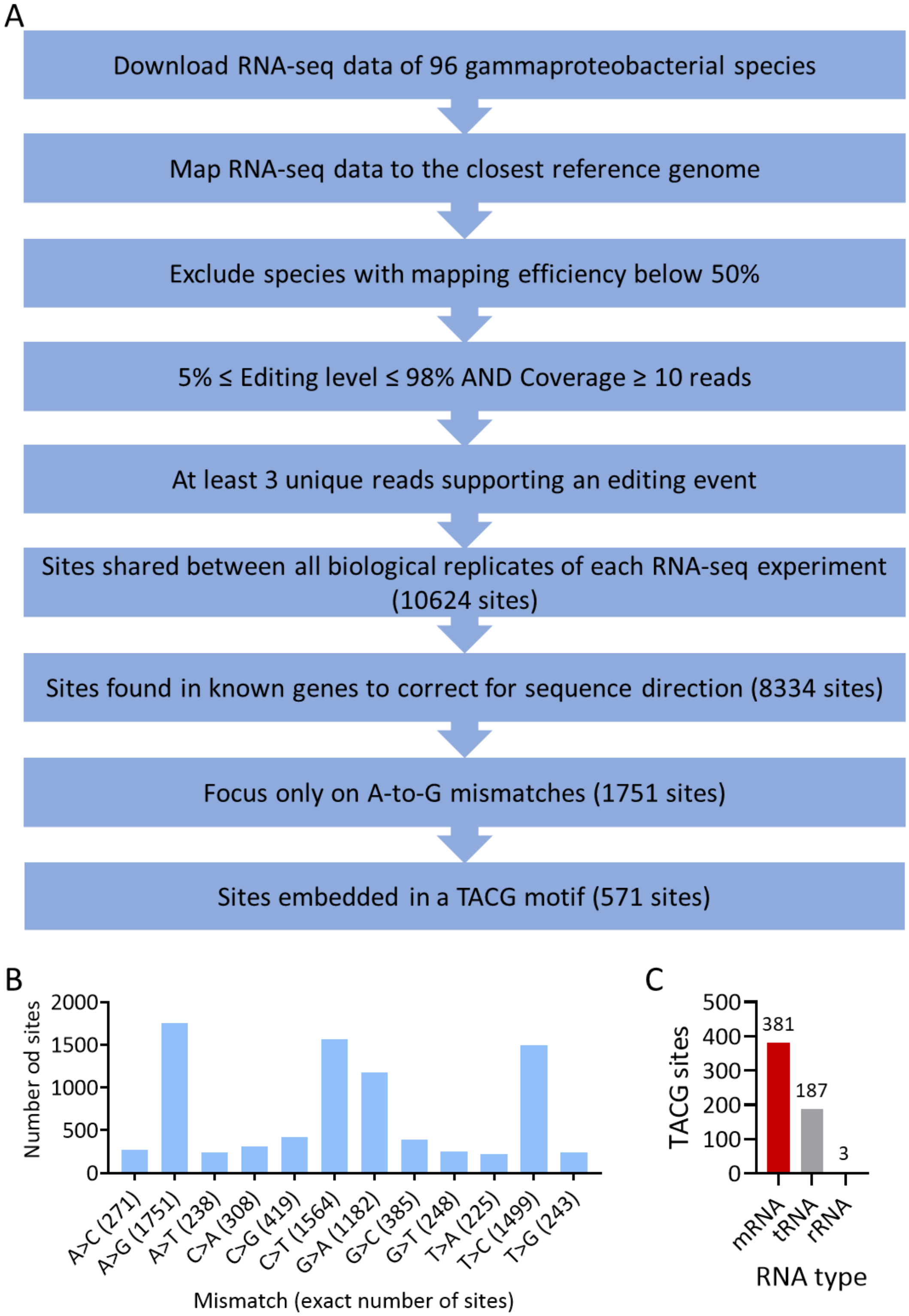

Supplementary Figure 2. A. Computational pipeline for RNA editing discovery. **B.** Distribution of all 12 mismatch possibilities in all sites identified within genes (total of 8334). **C.** Distribution of TACG-embedded RNA editing events shown in Figure 1C according to RNA type.

Score	Expect	Method	Identities	Positives	Gaps
159 bits(402)	5e-55	Compositional matrix adjust.	78/151(52%)	101/151(66%)	1/151(0%)

```
E. coli    17   FSHEYWMRHALTLAKRAWDEREVPVGAVLVHNNRVIGEGWNRPIGRHDPTAHAEIMALRQ  76
                F+ EYWM+ A   A RA +   EVPVGAV+V  N+VIG G+N PI   +DPTAHAEI A+R
A. baylyi       FNDEYWMQLAYEQAVRAAEHNEVPVGAVIVSQNKVIGSGYNAPITLNDPTAHAEIRAIRM  66

E. coli    77   GGLVMQNYRLI-DATLYVTLEPCVMCAGAMIHSRIGRVVFGARDAKTGAAGSLMDVLHHP  135
                    ++NYRL  DATLYVTLEPC MC GA++H+RI RVVF   + K G+  S   +L+
A. baylyi  7    ACESVKNYRLPEDATLYVTLEPCTMCVGALVHARIHRVVFATTEPKAGSLVSARQLLNMG  126

E. coli    136  GMNHRVEITEGILADECAALLSDFFRMRRQE  166
                  NH+  I  G +  +C+  LSDFFR RR++
A. baylyi  27   YYNHKFLIEHGCMQAQCSKQLSDFFRKRREQ  157
```

Supplementary Figure 3. Multiple sequence alignment by BLAST of TadA protein sequence from *E. coli* and *A. baylyi*. Position 64 in *E. coli* and position 54 in *A. baylyi*, which was mutated in this work, are marked in a red rectangle.

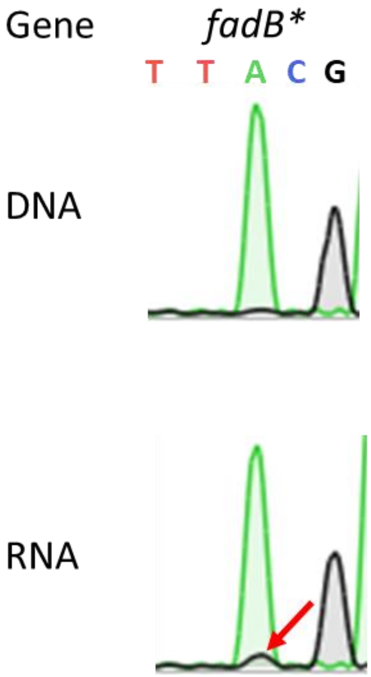

Supplementary Figure 4. A closeup of the gene *fadB* sequence from *V. alginolyticus* shown in Figure 1H. For better visualization, only the fluorescence signal intensities for A and G nucleotides are shown. Notice the small black peak in the RNA, but not the DNA sequence, representing the editing event.

A

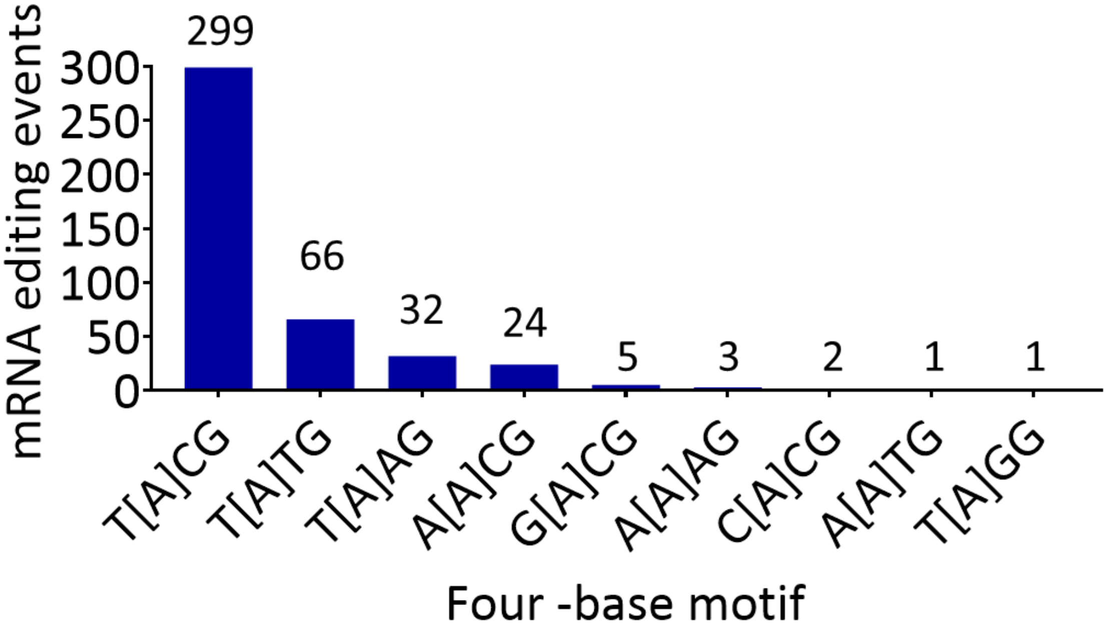

B

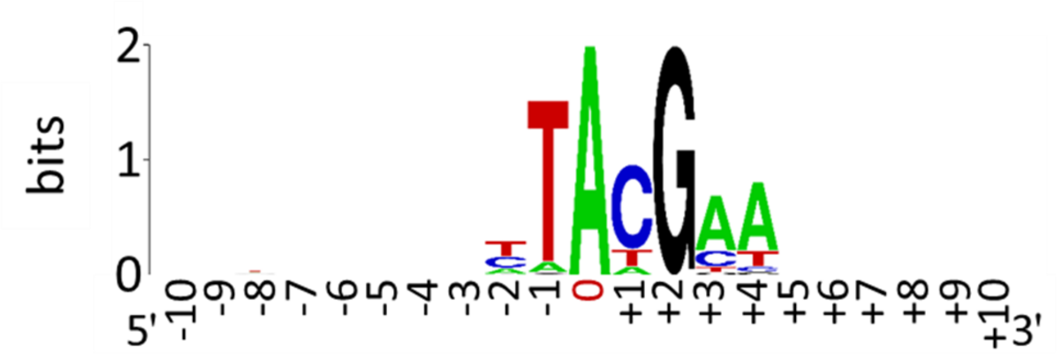

Supplementary Figure 5. The effect of the D54E substitution in TadA and TadA overexpression on RNA editing in *A. baylyi*. A. Four-base motif distribution of editing events detected in the $TadA^{D54E}$ overexpressing $TadA^{WT}$ from the pBTK402 plasmid. The edited adenosine is marked with brackets. **B.** WebLogo (48) analysis of the 433 mRNA editing events detected the $TadA^{D54E}$ strain overexpressing $TadA^{WT}$ from the pBTK402 plasmid.

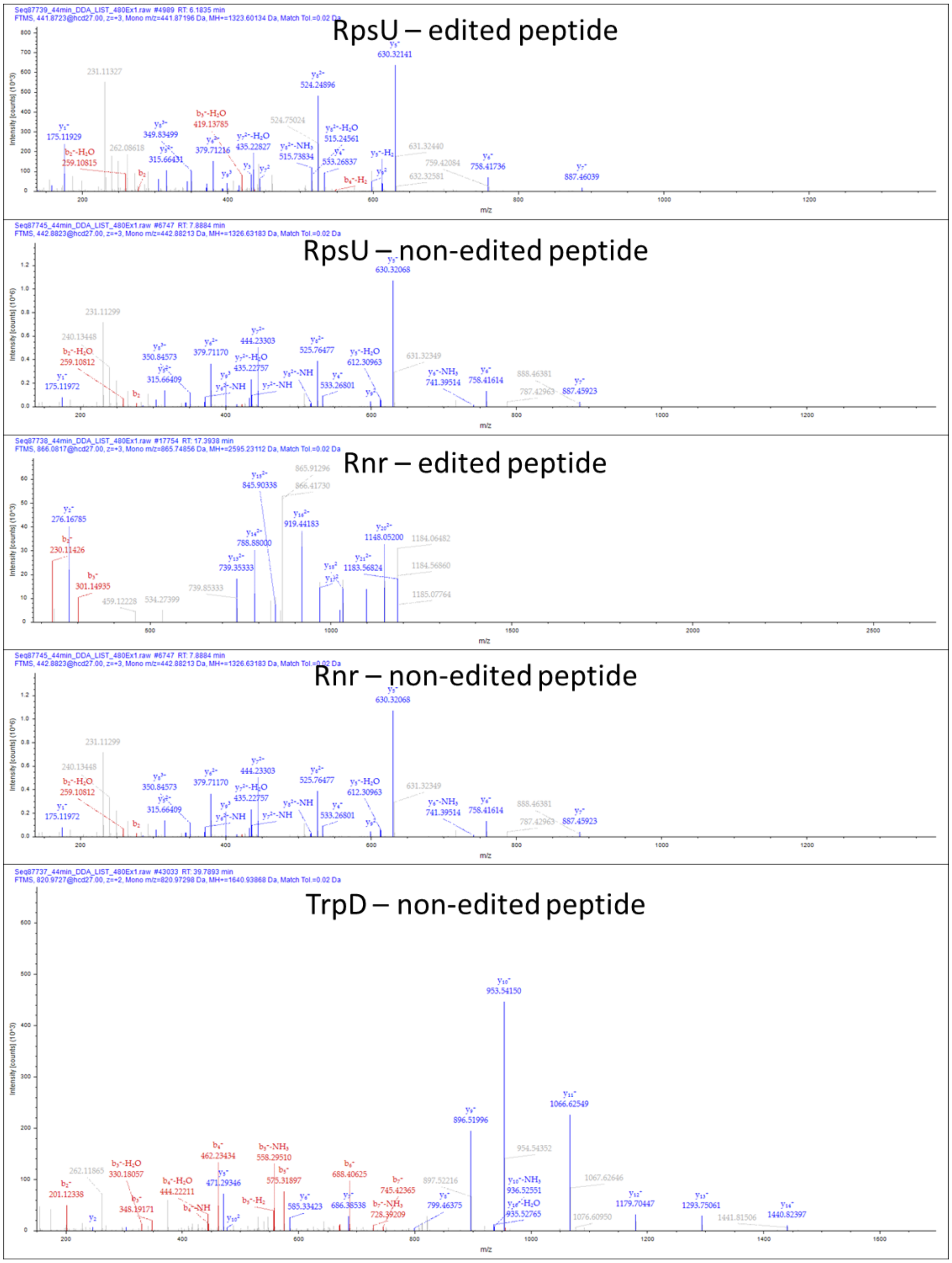

Supplementary Figure 6. A-to-I mRNA editing introduces protein isoforms in RpsU. A. The original MS/MS spectra of the edited and non-edited peptides of RpsU, Rnr and TrpD (only non-edited) in WT *A. baylyi*. The values of the expected and observed masses of peptides in the MS/MS analysis are found in Supplementary Table 18. All peptides were discovered with FDR ≤ 0.01.

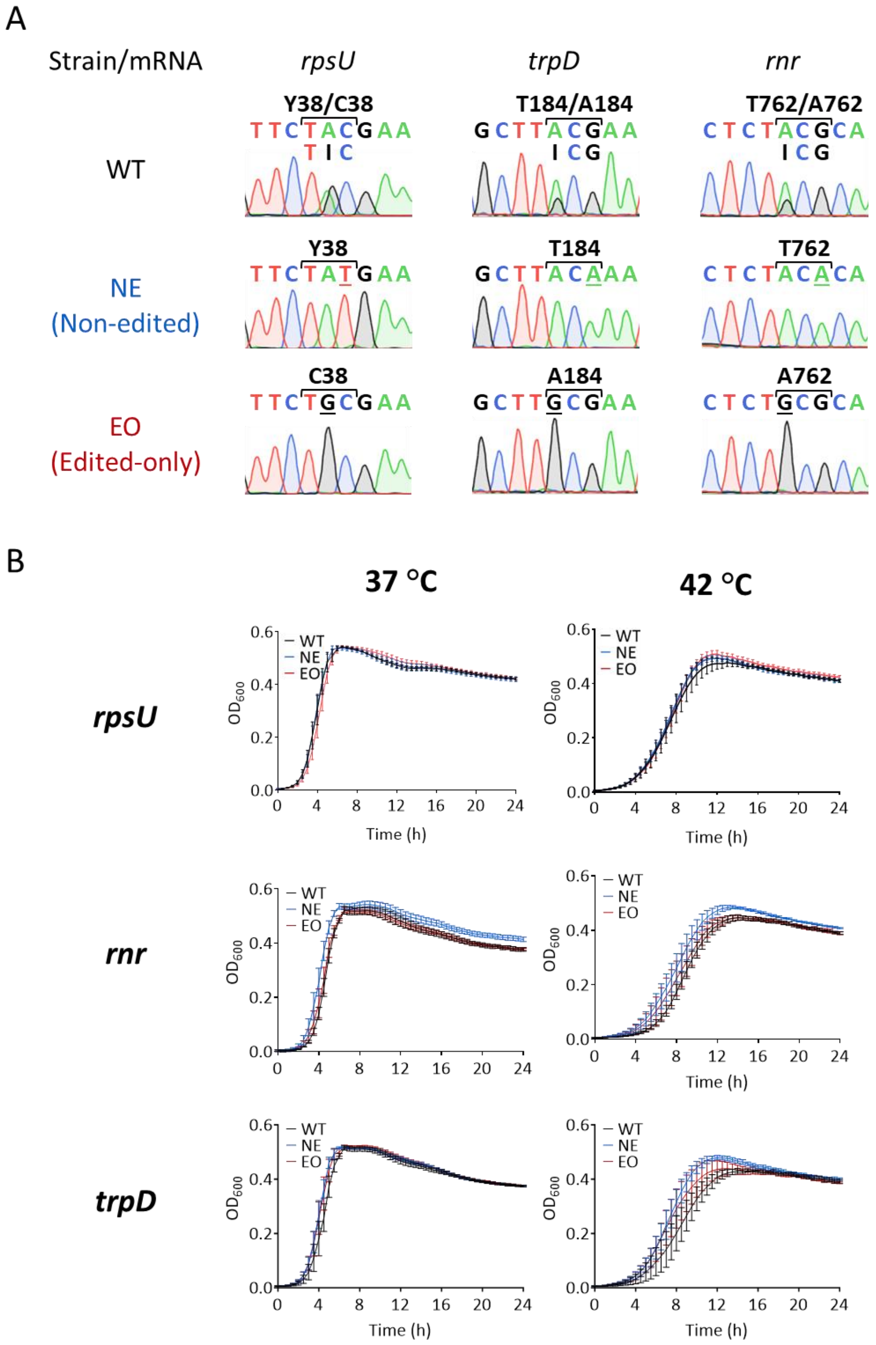

Supplementary Figure 7. Individual A-to-I mRNA editing events do not affect bacterial growth at different temperatures in LB. A. Sanger sequencing of three mRNAs of protein-encoding genes in the WT and mutant strains of *A. baylyi* harboring a single chromosomal mutation resulting in having only the non-edited or edited version of the designated gene/protein. **B.** Growth assays with the WT, non-edited only, and edited-only mutant strains of *A. baylyi* in LB at different temperatures. The mean and standard error of five biological replicates conducted on different days (N = 5), each with 28 technical replicates, are shown.

Supplementary Tables

Supplementary Table 1. Data of RNA-seq experiments of 96 bacterial species as found the NCBI SRA database.

Supplementary Table 2. Samples analyzed per species in this work.

Supplementary Table 3. A-to-G mismatches between the RNA-seq data and the reference genome of 72 bacterial species in annotated genes and the observed and expected frequencies of the four-motif used in Figure 1C and the chi square test within.

Supplementary Table 4. A-to-I mRNA editing events in RNA-seq samples of 64 bacterial species and their annotations.

Supplementary Table 5. Non-synonymous A-to-I mRNA editing events detected in RNA-seq samples of 64 bacterial species.

Supplementary Table 6. Synonymous A-to-I mRNA editing events detected in RNA-seq samples of 64 bacterial species.

Supplementary Table 7. TadA orthologs identified using tBLASTn in all examined species in this study.

Supplementary Table 8. A list of the partially conserved and conserved editing events shown in Figure 2D.

Supplementary Table 9. Amino acid identity at the position recoded by conserved editing events across hundreds to thousands of gammaproteobacterial species. The values used to construct Figure 2E.

Supplementary Table 10. Amino acid identity at the position recoded by conserved editing events across hundreds to thousands of gammaproteobacterial species. The values used to construct Supplementary Table 9.

Supplementary Table 11. Distribution and editing levels of the 381 mRNA editing events across different seven-base motif combinations.

Supplementary Table 12. DNA oligos used in the current study.

Supplementary Table 13. RNA editing events in the WT and TadA^{D54E} strains of *A. baylyi.* Ribosomal RNA-depleted RNA-seq identified eight mRNA editing events and two tRNA editing events (in transcripts of two *tRNA^{Arg2}* genes – locus tags ACIAD_RS14400 and ACIAD_RS14405).

Supplementary Table 14. RNA editing events in the TadA^{D54E} mutant strain supplemented with mRFP or TadA^{WT} from the pBTK402 plasmid. Ribosomal RNA-depleted RNA-seq identified a significant increase in all ten mRNA editing events in the TadA^{D54E} mutant when supplemented with the WT version of TadA from a plasmid compared to the same strain supplemented with a red fluorescent protein (mRFP).

Supplementary Table 15. Novel mRNA editing events and motif distribution in the TadA^{D54E} mutant strain supplemented with mRFP or TadA^{WT} from the pBTK402 plasmid. Ribosomal RNA-depleted RNA-seq identified a significant increase in all ten mRNA editing events in the TadA^{D54E} mutant when supplemented with the WT version of TadA from a plasmid compared to the same strain supplemented with a red fluorescent protein (mRFP).

Supplementary Table 16. The distribution of the 4-base-motif across 433 non-redundant A-to-I mRNA editing events upon TadA overexpression.

Supplementary Table 17. The distribution of the 7-base-motif across 433 non-redundant A-to-I mRNA editing events upon TadA overexpression.

Supplementary Table 18. Expected and observed masses of peptides in the MS/MS analysis of RpsU, Rnr, and TrpD shown in Supplementary Figure 5.
